# The relationship between rheological and textural properties of shrimp surimi adding starch and 3D printability based on principal component analysis

**DOI:** 10.1002/fsn3.2257

**Published:** 2021-03-25

**Authors:** Yanmo Pan, Qinxiu Sun, Yang Liu, Shuai Wei, Qiuyu Xia, Ouyang Zheng, Shucheng Liu, Hongwu Ji, Chujin Deng, Jiming Hao

**Affiliations:** ^1^ College of Food Science and Technology Guangdong Provincial Key Laboratory of Aquatic Product Processing and Safety Guangdong Province Engineering Laboratory for Marine Biological Products Guangdong Provincial Engineering Technology Research Center of Seafood Key Laboratory of Advanced Processing of Aquatic Product of Guangdong Higher Education Institution Guangdong Ocean University Zhanjiang China; ^2^ Collaborative Innovation Center of Seafood Deep Processing Dalian Polytechnic University Dalian China

**Keywords:** Rheological properties, Shrimp surimi, Starch, Textural properties, Three‐dimensional printing, Water properties

## Abstract

The three‐dimensional (3D) printing properties of pure shrimp surimi are poor and require improvement via the addition of other materials. The effects of the different amounts of potato starch, corn starch, tapioca starch, and cross‐linked starch (CLS) (0%, 3%, 6%, or 9%, respectively) on the 3D printing properties and material properties of white shrimp (*Litopenaeus vannamei*) surimi were evaluated in the present study. The results showed that the apparent viscosity, *G*′, and *G*'' of the samples were increased by adding 6% CLS, making it easy to extrude the sample from the nozzle and resulting in an improvement in the printing accuracy. In addition, after adding 6% CLS, the hardness, adhesiveness, and springiness of the sample were increased, which assist with maintaining the shape of the printed material and improve the printing stability of the sample. Moreover, the water holding capacity of shrimp surimi increased as a result of the addition of 6% CLS. In summary, 6% CLS can improve the material properties of shrimp surimi and make it more suitable for 3D printing.

## Graphical abstract

The three‐dimensional (3D) printing properties of pure shrimp surimi were poor. Adding 6% CLS could improve the rheological and textural properties of shrimp surimi. Shrimp surimi adding 6% CLS was more suitable for 3D printing.

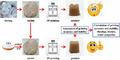

## 1 INTRODUCTION

White shrimp (*Litopenaeus vannamei*) is one of the three most cultured high‐yield shrimps in the world, showing fast growth, strong disease resistance, and significant economic benefits in breeding (Kureshy & Davis, 2002). Shrimp surimi is one of the high value‐added products of shrimp, which is popular because of its rich flavor and nutrition. However, the shape of shrimp surimi products is relatively simple at present, which limits their popularity and sales.

Three‐dimensional (3D) printing is a rapid prototyping technology for creating materials, usually by joining a layer upon layer, using data from digital model files (Dankar et al., 2018; Severini et al., 2016). Owing to its high accuracy, fast speed, and low cost, 3D printing is widely used in aerospace, biomedicine, architecture, food processing, and other fields (Murphy & Atala, 2014). With the improvement in the standard of living of people, consumers’ requirements for food are not only reflected in terms of the nutrition and taste but also in the attractive appearance of the food. Therefore, 3D printing technology is desirable in the food industry as it can produce geometric figures that are difficult to produce with traditional technology.

Despite the huge advantages of 3D printing technology, its research and application in the food processing industry have just begun. There are two basic requirements for the 3D printing of food materials: Raw food materials must have a certain liquidity, so that they are easy to extrude from the nozzle, and a level of supportability to help maintain the structure and shape of the object deposited on the platform after printing (Godoi et al., 2016). Although the 3D printing of many raw food materials has been studied, only 3D chocolate products are currently promoted commercially, while the properties of other raw food materials require adaptations. Shrimp surimi is a type of colloid that can form an elastic gel and has certain viscosity and fluidity. From the perspective of raw materials, shrimp surimi is suitable for 3D printing (Sánchez‐Alonso et al., 2014; Wang et al., 2018). However, in previous studies, we found that when using pure shrimp surimi for 3D printing, the output material was easy to break and the printed product was easy to collapse. This may have been caused by the higher moisture content of shrimp meat, which results in poor viscoelasticity and supportability. Starch is a commonly used auxiliary material in the production of surimi products (Dong et al., 2019). It not only increases the elasticity of the product by increasing water retention but also increases the yield and reduces the production costs. Starch has also been used in previous studies to effectively improve the 3D printing properties of *Scomberomorus niphonius* surimi (Dong et al., 2019), and mashed potatoes (Liu et al., 2018). However, few studies have been conducted regarding starch improving the 3D printing properties of minced shrimp.

Different types and amounts of starch influence the viscoelasticity and supportability of the material differently (Zheng et al., 2018). Therefore, starch with different chain: branch ratios (potato starch (PS), corn starch (CS), tapioca starch (TS), and modified starch (cross‐linked starch (CLS)) were added to shrimp surimi in different amounts (0%, 3%, 6%, and 9%, respectively), and the changes in the 3D printing properties, rheological properties, texture profile analysis (TPA), water distribution, and water holding capacity (WHC) of shrimp surimi were investigated to determine the most suitable starch for improving the 3D printing properties of shrimp surimi.

## 2 MATERIALS AND METHODS

### 2.1 Materials

Salt, cooking wine (rice wine), monosodium glutamate, and sugar were purchased from Wal‐Mart Stores (Zhanjiang, China). Potato starch, corn starch, tapioca starch, and cross‐linked starch (first grade) were purchased from Xin Liang Food Company (Xinxiang, China).

### 2.2 Preparation of samples

Fresh nonpregnant shrimps with an average weight of 15 ± 1 g were purchased from Happy Ocean (Zhanjiang, China) and immediately shipped to the laboratory in a tank with seawater. Then, the shrimps were stunned in an ice bath, beheaded, and shelled, before lining and washing under low temperature conditions (4°C). The shrimp muscles were chopped for 5 min in a mixer machine (MQ785, Braun, Germany). Then, the minced shrimp meat was mixed with iced water at a ratio of 1:5 (w/w) for three washing cycles. The shrimp paste was then wrapped in three layers of gauze for manual dewatering. Then, 3% (w/w) salt was added to the shrimp, which was then chopped for 2 min. Next, 3% (w/w) cooking wine, 1% (w/w) monosodium glutamate, and 1% (w/w) sugar were added to the shrimp, which was then chopped for 2 min. Finally, the shrimp surimi was divided into 13 equal portions, among which, 12 portions of shrimp surimi were chopped for a further 2 min, with different amounts (3%, 6%, and 9%) of PS, CS, TS, and CLS added. The final portion of shrimp surimi was chopped for another 2 min without any starch and was used as a control. The temperature during the preparation process was maintained at 2–5°C. The shrimp surimi was printed immediately once it was prepared.

### 2.3 Printing accuracy and stability

In this study, a syringe‐type fused deposition modeling 3D food printer (FOODBOT E1, Shinnove, Zhejiang, China) was used. The shrimp surimi with different starch additions were loaded into a 3D printed extrusion barrel, the 3D printer nozzle diameter was 1.20 mm, the printing speed was set to 30 mm/s, the printing height was set to 2 mm, and the printer barrel temperature was set to 25°C for printing. To conveniently measure the printing accuracy and stability of the printed product, a cube model with a side length of 20 mm was designed. The 3D printing properties of the shrimp surimi were evaluated by assessing their appearance during printing as well as the printing accuracy and stability of the 3D printed cubes. The printing accuracy of shrimp surimi was measured after printing was completed. For the printing stability measurement, the printed products were placed at room temperature (25°C) for 60 min, and the length and height of the sample after printing for 0 and 60 min were measured using Vernier callipers. The printing accuracy and stability were calculated as follows:

$$\text{Printing accuracy}\;\left(\%\right)=\left(1‐\left|\frac{L_{s}‐L_{m}}{L_{m}}\right|\right)\times 100\%$$

$$\text{Printing stability}\;\left(\%\right)=\frac{H_{60\min}}{H_{0\min}}\times 100\%$$

where *L_s_* represents the side length of the printed sample (mm); *L*_m_ represents the side length of the model (mm); *H*_0min_ represents the height (mm) of the sample stored for 0 min; and *H*_60min_ represents the height (mm) of the sample stored for 60 min.

### 2.4 Rheological properties analysis

The rheological properties of the mixed shrimp surimi at different starch levels were determined by an advanced modular rheometer (HAAKE MARS Ⅲ, Thermo Fisher Scientific, USA) with a parallel plate (diameter = 20 mm). The gap between the two plates was set to 1.0 mm. For the determination of steady shear apparent viscosity, the shear rate was ramped from 0.1 to 100 s^‐1^. After loading, the sample was allowed to rest for 2 min at 25°C.

The dynamic viscoelasticity properties were measured by a small amplitude oscillatory frequency sweep mode. The frequency oscillated from 0.1 to 10 Hz, and all measurements were performed within the identified linear viscoelastic region and made at 0.1% strain. The storage modulus (*G*′), loss modulus (*G*''), and loss tangent (tan δ, = *G*''/*G*') were recorded (Liu et al., 2018; Wang et al., 2018).

### 2.5 Texture profile analysis

The texture properties of the shrimp surimi samples were analyzed by TPA using a texture analyzer (TMS‐Pro, FTC, USA), and the detection indexes included hardness, adhesiveness, and springiness. The instrument was calibrated with a 1 kg load cell, and the test probe was a round cake with a diameter of 38 mm. Sample test parameters were as follows: The height of the probe rising to the sample surface was 30 mm, the compression deformation was 50%, the detection speed was 60 mm/min, and the trigger force was 0.5 N.

### 2.6 Water distribution analysis

The transverse relaxation time (*T*_2_) of shrimp surimi with different starch additions was measured with a low‐field nuclear magnetic resonance (LF‐NMR) instrument (NMI20‐060H‐I, Niumag Electric Corporation, Shanghai, China). The resonance frequency was set to 22.6 MHz, and the magnetic field strength of the instrument was set to 0.47 T. Then, 10 g of shrimp surimi was placed in a 35 mm‐diameter plastic dish, and the transverse relaxation curve was derived using the Carr–Purcell–Meiboom–Gill pulse sequence mode. The NMR imaging (MRI) of shrimp surimi samples was measured by the same LF‐NMR analyzer, according to the methods of Zhang et al., (2019) with minor modifications. Proton density images of samples were obtained by a spin‐echo imaging sequence. Four‐layer cross‐sections were selected for imaging in the middle area of the sample. The thickness of each layer was 1.75 mm, and the interval between layers was 0.65 mm. Then, the scan protocol was set to obtain images according to the following parameters: The scanning frequency was 21.12 MHz, repetition time was 2,200 ms, echo time was 20.00 ms, and average was 4. The gray level images were processed by Niumag NMR Image Processing software (Version 3.0, Niumag Corporation, Shanghai, China) to obtain pseudocolor images.

### 2.7 Water holding capacity of shrimp surimi

The WHC of the shrimp surimi samples was determined using centrifugation (Zhang et al., 2016). About 10 g (*W*_1_, g) of shrimp surimi sample was placed in a 50 ml centrifuge tube and centrifuged at 10,000 × *g* for 10 min at 4°C with a refrigerated centrifuge (3K‐15, Sigma, Germany). After centrifugation, the surface water of the shrimp surimi was absorbed by the filter paper, and the weight of the sample was accurately weighed (*W*_2_, g). The WHC was calculated with the following equation:

$$\text{WHC}\;\left(\%\right)=\frac{W_{2}}{W_{1}}\times 100\%$$

### 2.8 Water content of shrimp surimi

The water content of the shrimp surimi was determined by drying samples in a drying oven (DHG‐9420A, Yiheng Corporation, Shanghai, China) at 105°C until a constant weight was achieved. The water content was calculated as follows:

$$\text{Water content}\;\left(\%\right)=\frac{W_{s}‐W_{d}}{W_{s}}\times 100\%$$

where *W_s_* represents the sample weight (g); *W*_d_ represents the dried sample weight (g).

### 2.9 Statistical analysis

Data were expressed as mean ± standard deviation and analyzed by the general linear model program of JMP software (Version Pro 13, SAS Institute Inc., Cary, NC, USA). The significance between means was determined by two‐way analysis of variance and Tukey's multiple comparison method with a confidence interval of 95% (*p* <.05). Principal component analysis (PCA) was performed by SPSS 22.0 (analysis software, USA). Three batches of (replicated) shrimp surimi were produced. For each batch of samples, all measurements were performed in triplicate. In particular, the TPA experiment was repeated five times.

## 3 RESULTS AND DISCUSSION

### 3.1 Printing accuracy and stability analysis

**FIGURE 1:**
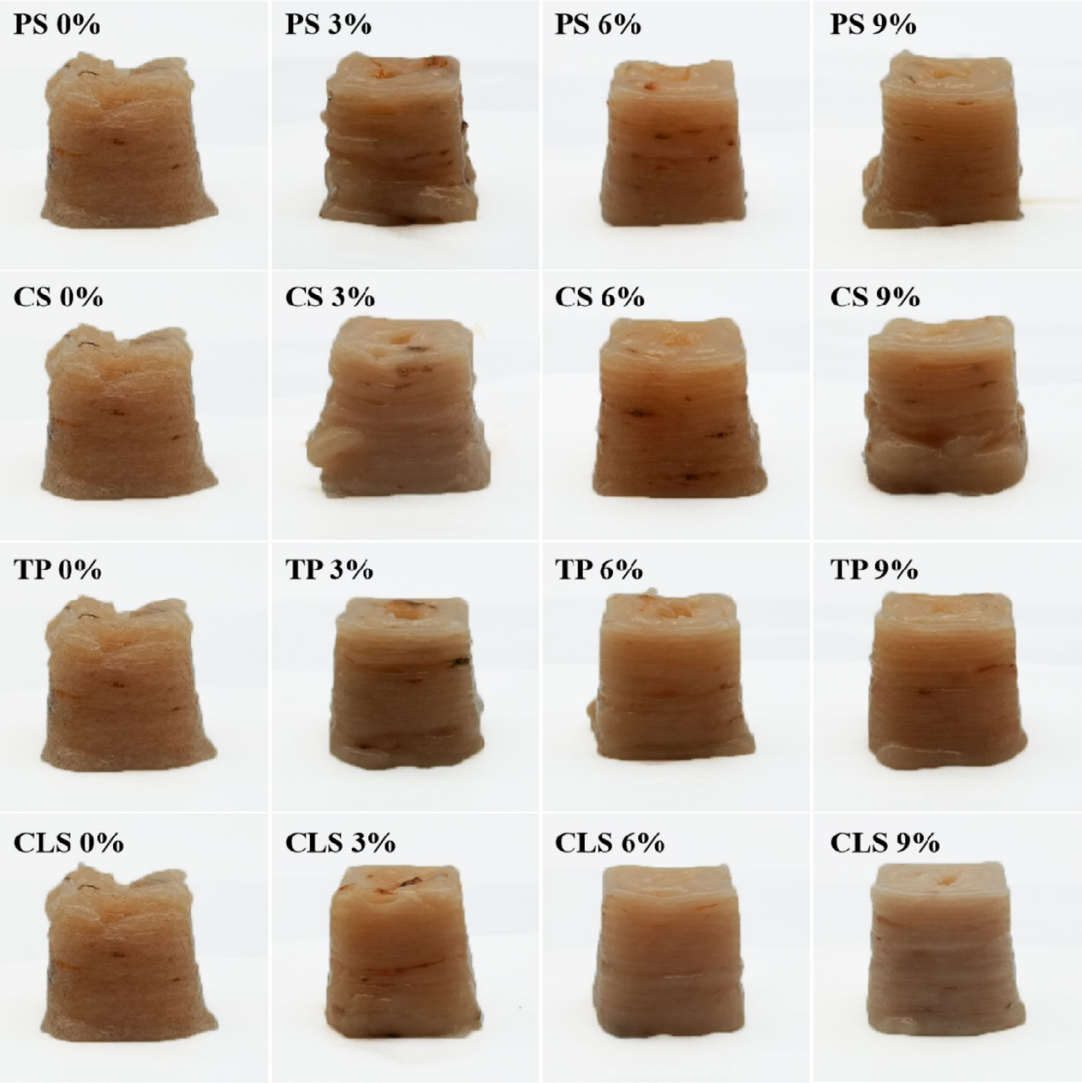
Photographs of 3D printing shrimp surimi with different types of starch added at 0 min, PS, potato starch; CS, corn starch; TS, tapioca starch; CLS, cross‐linked starch

**FIGURE 2:**
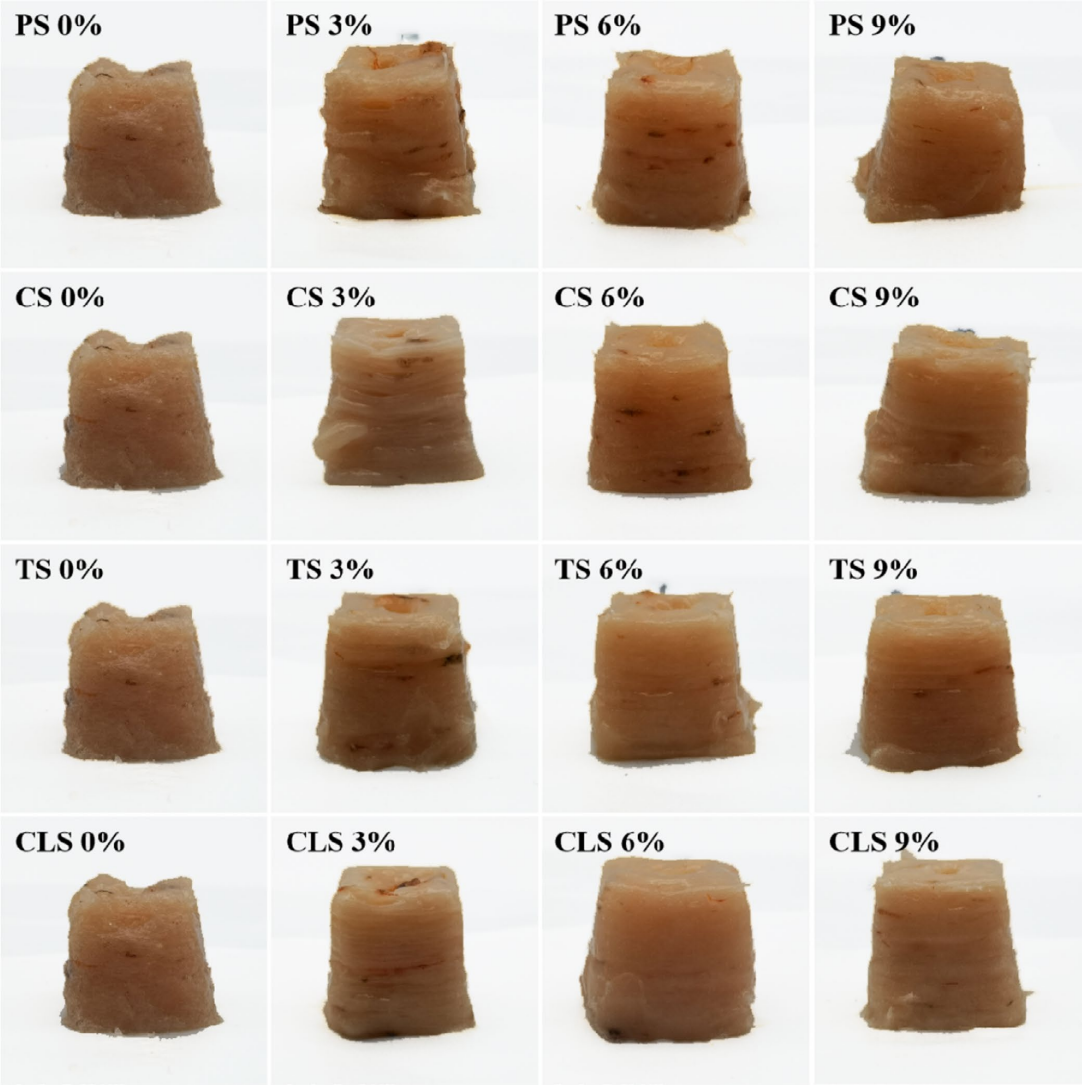
Photographs of 3D printing shrimp surimi with different types of starch added at 60 min, PS, potato starch; CS, corn starch; TS, tapioca starch; CLS, cross‐linked starch

The 3D printing accuracy and stability are important indicators for evaluating the quality of 3D printing products (Liu et al., 2017). The visual appearance of the 3D structure placed 0 and 60 min after printing is shown in Figure 1 and Figure 2, respectively. Breakage, collapse, and poor printing accuracy were observed in the 3D structure of control samples (without starch added). This may be due to the high moisture content, water mobility, and low viscoelasticity of shrimp surimi (Wang et al., 2018). Adding a small amount of starch (3%) improved the printing accuracy of shrimp surimi, but there still existed uneven outflow of material and collapse. The 3D printing structures of shrimp surimi were improved by adding an appropriate amount of starch (6%), resulting in a smooth appearance, high printing accuracy, and good match with the virtual model. This may be explained by the strong gel binding capacity of starch, which has a degree of water absorption and expansion properties, thereby increasing the supportability and viscoelasticity of shrimp surimi (Dong et al., 2019). However, the printing accuracy of the sample decreased when too much starch (9%) was added, which may be due to the deterioration of the fluidity of the material, making the sample eject suddenly, and making it difficult to squeeze out the nozzle or even block the nozzle. For the samples with the same amount of starch added, the printing accuracy of the CLS sample was the highest, followed by TS, PS, and CS samples (*p* <.05). This may be because CLS is a modified starch, which has higher viscosity and stability than the original starch (Eliasson, 2007), thus, making the material squeeze out from the nozzle smoothly and increasing the adhesion between layers. Amylopectin has higher adhesion and water absorption than amylose (Hunt et al., 2009). TS is rich in amylopectin, while PS and CS are rich in amylose. Therefore, the TS group has higher adhesion and water absorption than the PS and CS group, which increased the continuity and layering of the material during printing, improved the material's fragile filament and collapse problems, and increased the printing accuracy and stability of the surimi samples.

By comparing Figure 1 and Figure 2, it can be observed that the printed products showed different degrees of collapse after leaving to stand for 60 min. As illustrated by Table 1, for the samples with the same type of starch added, the printing stability of the samples gradually increased with the increase of starch content. This may be because the increase of starch content gradually increased the WHC and hardness of shrimp surimi, thereby increasing the supportability and stability of the surimi. For the samples with the same amount of starch added, the printing stability of the CLS sample was the highest, followed by the TS, PS, and CS samples (*p* <.05). This may be due to the high degree of cross‐linking of CLS, which can increase the stability of the protein internal network structure. In addition, modified starch has inherent physical and chemical advantages such as good solubility in cold water, high water resistance, good mechanical shear resistance, and good freeze‐thaw stability, which can increase the stability of materials (Kou & Gao, 2019). TS contains more amylopectin than PS and CS, resulting in stronger viscosity, making shrimp surimi form a stronger gel by fully locking the water in the gel network structure (Yang & Park, 1998); thus, the TS group had higher WHC than the PS and CS groups. Therefore, the shrimp surimi with added CLS and TS had better supportability and higher printing stability than the other samples. In summary, the addition of 6% CLS effectively improved the 3D printing properties of shrimp surimi.

**TABLE 1:** Effects of different types of added starch on the printing accuracy and stability of three‐dimensional shrimp surimi structure

Index	Addition (%)	PS	CS	TS	CLS
Printing accuracy (%)	0	87.00 ± 0.85^g^	87.00 ± 0.85^g^	87.00 ± 0.85^g^	87.00 ± 0.85^g^
	3	87.31 ± 0.59^fg^	87.16 ± 0.23^fg^	88.97 ± 0.52^ef^	91.30 ± 0.79^d^
	6	93.53 ± 0.26^bc^	93.10 ± 1.00^bcd^	94.74 ± 0.03^b^	96.75 ± 0.04^a^
	9	91.56 ± 0.67^d^	89.31 ± 0.59^e^	92.10 ± 0.72 cd	94.74 ± 0.08^b^
Printing stability (%)	0	90.23 ± 0.41^k^	90.23 ± 0.41^k^	90.23 ± 0.41^k^	90.23 ± 0.41^k^
	3	92.30 ± 0.17^ij^	91.67 ± 0.36^j^	93.04 ± 0.24^hi^	93.65 ± 0.42^h^
	6	95.30 ± 0.09^fg^	94.90 ± 0.22^g^	97.06 ± 0.31 cd	98.44 ± 0.34^ab^
	9	96.49 ± 0.18^de^	95.91 ± 0.37^ef^	97.69 ± 0.16^bc^	98.72 ± 0.08^a^

Note

Values are means ± standard deviations. Values with different superscript letters in the same indicator are significantly different (*p* <.05).

Abbreviation: CLS, cross‐linked starch; CS, corn starch; PS, potato starch; TS, tapioca starch.

### 3.2 Rheological properties of shrimp surimi

#### 3.2.1 The static shear rheology of shrimp surimi

**FIGURE 3:**
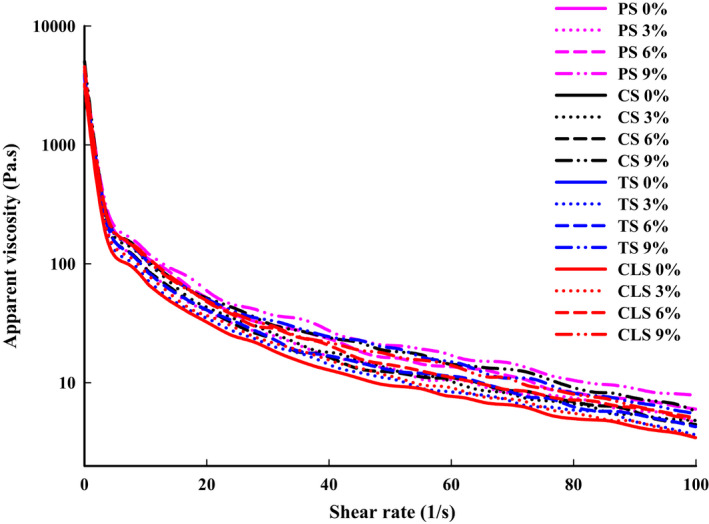
Apparent viscosity of shrimp surimi with different types of starch added, PS, potato starch; CS, corn starch; TS, tapioca starch; CLS, cross‐linked starch

During the 3D printing process, the rheological properties of the material affect the continuity and stackability of the output. Therefore, rheological properties are important indicators that affect 3D printing (Yang et al., 2018). The apparent viscosity curve of the shrimp surimi is shown in Figure 3. The apparent viscosity decreased with the increase of shear rate, and had high apparent viscosity at low shear rate, indicating that all shrimp surimis were pseudoplastic fluids with shear thinning. Previous studies have identified that 3D printed food materials should be pseudoplastic fluids with shear‐thinning properties to make samples easy to squeeze and shape (Azam et al., 2018). In addition, as the amount of starch increased, the apparent viscosity gradually increased. This may be due to that starch granules absorbed water and swelled during the collapse process and finally formed a denser network structure (Liu et al., 2018). Whittenberger and Nutting (2002) also reported that the gel strength and viscosity of PS paste increased directly with starch concentration increase. For the samples with the same amount of starch added, the apparent viscosity of the TS group was highest, followed by the CLS, PS, and CS groups (*p* <.05). This might be related to the different effects of starch on the gel properties of shrimp surimi as a result of the different ratios and types of branched and straight chains in starch granules (Hunt et al., 2009). The apparent viscosity of the CLS samples was neither the highest nor the lowest, which may be because the cross‐linking of starch has two opposite effects on starch paste through two main mechanisms (Steeneken, 1989). On the one hand, cross‐linking reduces the loss of soluble substances (amylose and small amylopectin), thereby improving the viscosity of the paste; on the other hand, it can inhibit the expansion force of the particles, thereby reducing the viscosity of the starch paste. The formation of cross‐linked covalent bonds between amylose and amylopectin chains can enhance the swelling of starch granules, thereby reducing the breakage of the paste under mechanical shear (Wongsagonsup et al., 2014). Combined with the visual appearance of 3D products (Figure 1), it was observed that the 3D printability of the sample was not improved with a larger starch content. This may be because for 3D printing materials, their apparent viscosity should be low enough for the material to be easily extruded from the nozzle, and high enough for bonding between layers (Liu et al., 2018).

#### 3.2.2 Dynamic frequency rheology of shrimp surimi

**FIGURE 4:**
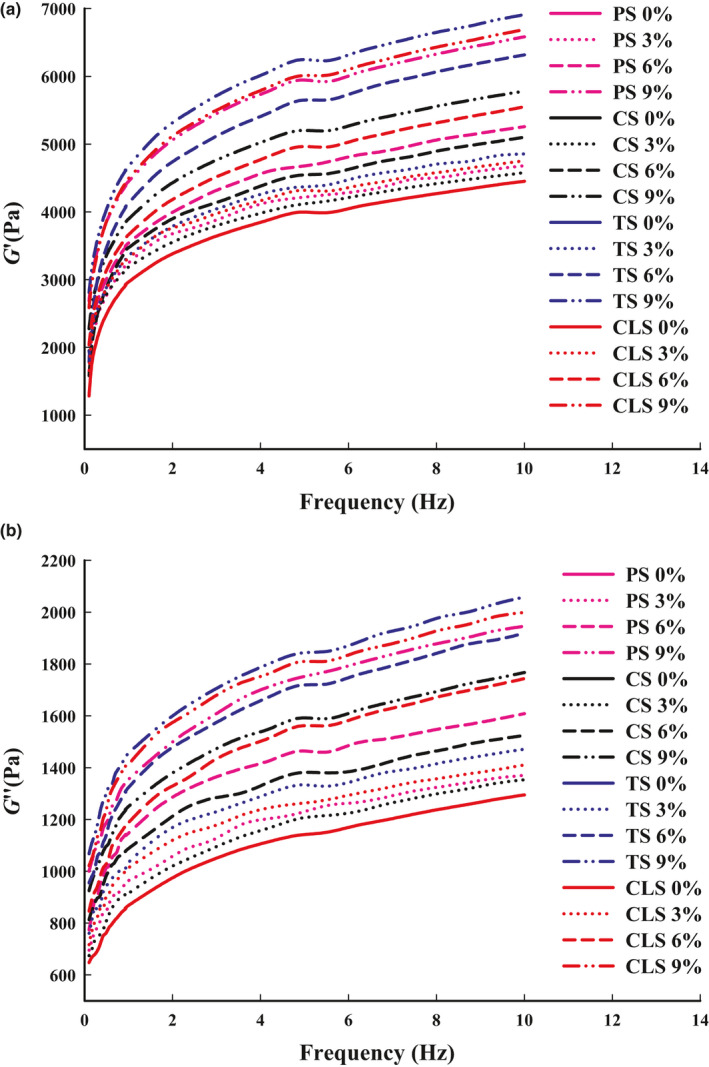
Storage modulus (*G*′) (a) and loss modulus (*G*'') (b) of shrimp surimi with different types of starch added. PS, potato starch; CS, corn starch; TS, tapioca starch; CLS, cross‐linked starch

**FIGURE 5:**
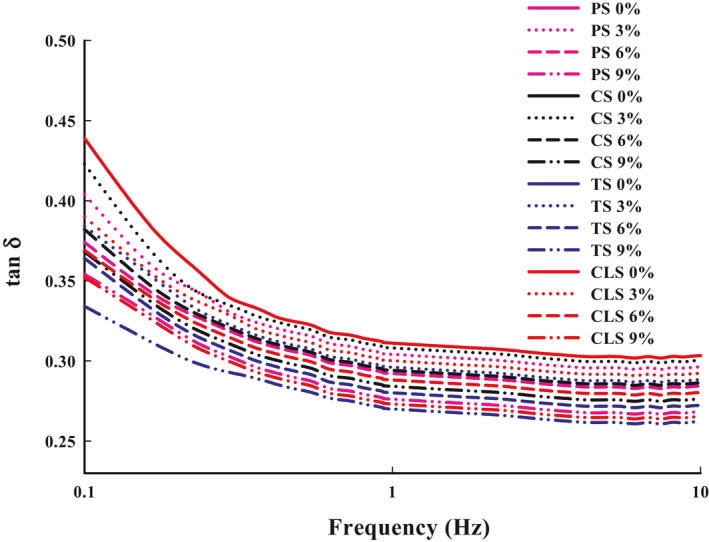
Loss tangent (tan δ, = *G*''/*G*') of shrimp surimi with different types of starch added. PS, potato starch; CS, corn starch; TS, tapioca starch; CLS, cross‐linked starch

The rheological properties of the material directly affect the printing effects of the product, which are mainly reflected in whether printing broken. The *G*' reflects the elastic solid‐state behavior of the sample, the *G*'' measures the viscous response, while the tan δ can be used as a characterization parameter to display different viscoelastic behaviors. In light of the shrimp surimi viscoelastic properties, the *G*' was higher than the *G*'' in the linear viscoelastic region (Figure 4a and b), suggesting its potential to form elastic gel or gel‐like structures. In addition, Figure 5 shows that the tan δ values of all shrimp surimi were lower than 1, indicating that the material exhibits solid‐like characteristics with poor fluidity (Fischer & Windhab, 2011). The changes of *G*' and *G*" of all the samples were similar, both *G*' and *G*" gradually increased with the increase of oscillation frequency, leading to the increased internal friction of the material. In addition, at any oscillation frequency, *G*' and *G*" increased continuously with the increase of starch content. Chen et al., (2018) thought that the increasing of *G*' may be because of the closely packed matrix caused by the swelling of starch granules. For the samples with the same amount of starch added, *G*' and *G*" of the TS group were the highest, followed by the CLS, PS, and CS groups. Combined with the properties of 3D printing, it can be seen that the *G*' and *G*'' of pure shrimp surimi were too small, making them prone to breakage and poor stacking performance during printing. The addition of starch increased the *G*' and *G*" of the samples, and appropriate *G'* and *G''* facilitates the flow of material from the nozzle and increases adhesion between the layers of the printed material (Wang et al., 2016), thus, improving the 3D printing properties.

### 3.3 Texture profile analysis *of* shrimp surimi

Texture properties affect the supportability of materials, which affects the ability of 3D printed products to maintain their shape. The texture properties of the shrimp surimi are shown in Table 2. The hardness, adhesiveness, and springiness of the samples were measured in the present study, which are important texture indicators that affect the 3D printing properties of shrimp surimi. The hardness, which refers to the force required when the sample just begins to deform (Severini et al., 2018), of all samples increased with the increase of starch content (*p* <.05). The hardness of the control group was 0.70 N, while that of shrimp surimi with 9% PS, CS, TS, and CLS increased to 0.94, 0.88, 0.98, and 1.01 N, respectively, which may be explained by starch absorbed with the extra moisture increasing the hardness of the raw materials. Starch can expand by absorbing water to stabilize the protein network structure inside the shrimp surimi and increase its hardness. Among the samples with the same starch content, the CLS group had the highest hardness, followed by the TS, PS, and CS groups (*p* <.05), which corresponds to the results of the WHC. Since native starch is linked together by hydrogen bonds, which is extremely unstable under acidic, high temperature and shear conditions. After the introduction of cross‐linking agents, the physicochemical structure of starch granules can be strengthened by covalently connecting the hydroxyl groups of adjacent polymers (Sang et al., 2007). In addition, the high WHC of the sample also contributed to the increase of the hardness of samples (Zhang, Fang, et al., 2013). Combined with the 3D printing properties, it can be concluded that high hardness is conducive to maintaining the original shape of the product, thus, improving the printing stability of the product. Although the sample with 9% starch had the greatest hardness, it would clog the printer nozzles, causing uneven outflow of material and the sudden spraying of large amounts of material. Therefore, the sample with 6% added starch has appropriate hardness and better printing effects.

**TABLE 2:** Effects of different types of added starch on the TPA of shrimp surimi

Index	Addition (%)	PS	CS	TS	CLS
Hardness (*N*)	0	0.70 ± 0.02^g^	0.70 ± 0.02^g^	0.70 ± 0.02^g^	0.70 ± 0.02^g^
	3	0.71 ± 0.02^fg^	0.71 ± 0.02^fg^	0.74 ± 0.02^fg^	0.78 ± 0.04^ef^
	6	0.84 ± 0.04^de^	0.82 ± 0.04^de^	0.88 ± 0.04 cd	0.92 ± 0.04^bc^
	9	0.94 ± 0.02^abc^	0.88 ± 0.02 cd	0.98 ± 0.04^ab^	1.01 ± 0.05^a^
Adhesiveness (*N*·mm)	0	0.95 ± 0.04^g^	0.95 ± 0.04^g^	0.95 ± 0.04^g^	0.95 ± 0.04^g^
	3	1.47 ± 0.24^efg^	1.19 ± 0.22^fg^	1.85 ± 0.34^def^	1.59 ± 0.33^efg^
	6	2.16 ± 0.39^de^	1.60 ± 0.25^efg^	3.07 ± 0.17^bc^	2.38 ± 0.27 cd
	9	3.16 ± 0.60^ab^	2.91 ± 0.58^bc^	3.83 ± 0.44^a^	3.41 ± 0.26^ab^
Springiness (mm)	0	1.96 ± 0.04^h^	1.96 ± 0.04^h^	1.96 ± 0.04^h^	1.96 ± 0.04^h^
	3	3.10 ± 0.27^fg^	2.10 ± 0.32^gh^	3.54 ± 0.76^f^	3.41 ± 0.26^f^
	6	3.83 ± 0.27^ef^	3.58 ± 0.73^f^	5.84 ± 0.64 cd	4.75 ± 0.72^de^
	9	6.42 ± 0.80^bc^	5.08 ± 0.45^d^	7.99 ± 0.18^a^	6.99 ± 0.78^ab^

Note

Values are means ± standard deviations. Values with different superscript letters in the same indicator are significantly different (*p* <.05).

Abbreviation: CLS, cross‐linked starch; CS, corn starch; PS, potato starch; TS, tapioca starch.

Adhesiveness refers to the work done by the probe against the resistance caused by the adhesion of the sample, which reflects the attraction between the sample and the material in contact with the sample (Jonsson et al., 2015). With the increase of starch content, the adhesiveness of the sample gradually increased. Among the samples with the same amount of starch added, the adhesiveness of the TS group was significantly higher than that of any other groups, followed by the CLS, PS, and CS groups (*p* <.05). The adhesiveness of the 9% TS group was 3.83 N·mm, which was four times higher than that of the control group (0.95 N·mm). This may be because TS is amylopectin, which has a high viscosity and can enhance the adhesion of shrimp surimi (Zhou et al., 2015). Combined with the 3D printing properties, it can be concluded that proper adhesiveness is beneficial for adhesion between printed sample layers.

Springiness is the ability of a sample to return to its original shape after deformation (Yang et al., 2018). As the starch content increased, the springiness of the sample gradually increased. The change of springiness in shrimp surimi was similar to that of adhesiveness. It was found that the springiness of the 9% PS, CS, TS, and CLS groups was 6.42, 5.08, 7.99, and 6.99 mm, respectively, which was more than 1.5 times higher than that of the control group (1.96 mm). This may be because the starch forms a starch–protein–water composite network structure with the water and protein in the shrimp surimi during the chopping process and cross‐linked with polysaccharides to form a larger and more complex network structure, thereby enhancing the springiness of the shrimp surimi (Kong et al., 2016).

The results of TPA showed that the types and content of starch directly affected the texture properties of the shrimp surimi‐starch gel system. The proper texture properties of samples should not only be easy to extrude but also show a certain load‐bearing capacity to maintain the original shape of printing materials (Azam et al., 2018; Salmeron, 2006; Yang, Zhang, Prakash, et al., 2018). Therefore, in combination with the 3D printing effects, it can be concluded that proper hardness, adhesiveness, and springiness are beneficial to the extrusion molding of shrimp surimi and the adhesion between the layers, thereby improving the printing effect of shrimp surimi.

### 3.4 Water distribution of shrimp surimi

**FIGURE 6:**
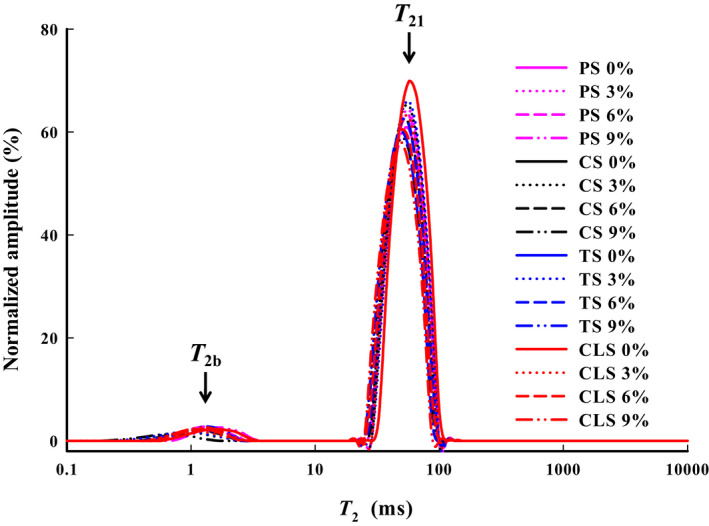
LF‐NMR signal for shrimp surimi with different types of starch added, PS, potato starch; CS, corn starch; TS, tapioca starch; CLS, cross‐linked starch

LF‐NMR analysis, as a new, nondestructive testing method, can reflect changes in the water state, distribution, and composition of food (Ahmad et al., 2007). The change of water distribution directly affects the microstructure of the system and the modelling effect of 3D printed samples. As shown in Figure 6, two peaks were observed in the shrimp surimi LF‐NMR curves, indicating that the shrimp surimi proteins limit the water mobility with different magnitudes. Water population *T*_2b_ (0–10 ms) represents the lower amount of bound water, which is closely bonded within the macromolecules, and water population *T*_21_ (10–100 ms) reflects the water trapped within the gel microstructure (immobilized water).

Table 3 shows the effects of starch on the *T*_2_ and amplitude peak area ratio (*A*_2_) of the two water populations. Figure 6 and Table 3 show that, for the same type of starch, with an increase in starch content, the *T*_2_ of the samples shifts to the left (short relaxation time direction), *A*_21_ gradually decreases, and *A*_2b_ gradually increases. These results indicate that the addition of starch increases the ability of shrimp surimi to bind water. This is mainly due to the fact that starch molecules contain a large number of hydrophilic groups, which can promote the hydrogen bonding of starch and water, increase the stability of bound water, thus, reduce the *T*_2b_ and *T*_21_ (Goh et al., 2009). In addition, the water molecules reoriented more slowly at higher starch concentrations, enhancing the chemical exchange between starch hydroxyl protons and water protons (Yakubu et al., 1993). For the group with the same starch content added, the CLS group had the largest *A*_2b_, followed by the TS, CS, and PS added groups (*p* <.05). This might be due to the strong swelling ability of modified starch, which can be fully filled in the structure of shrimp surimi, increasing the ability of shrimp surimi to hold water. Both CS and PS content more amylose. Due to their tightly closed helical structure, they are not conducive to the formation of strong intramolecular hydrogen bonds with water molecules or to combining with water molecules. TS is rich in amylopectin, which is relatively open owing to its high degree of branching and is good for hydrogen bonding with water molecules (Zhang et al., 2013). This result corresponds to that of WHC, indicating that the addition of starch mainly increases the WHC of shrimp surimi by improving its ability to bind water.

**TABLE 3:** Effects of different types of added starch on the water state distributions of shrimp surimi

Index	Addition (%)	PS	CS	TS	CLS
*T*_2b_	0	1.75 ± 0.01^a^	1.75 ± 0.01^a^	1.75 ± 0.01^a^	1.75 ± 0.01^a^
	3	1.52 ± 0.02^b^	1.32 ± 0.01^c^	1.32 ± 0.01^c^	1.32 ± 0.02^c^
	6	1.32 ± 0.01^c^	1.15 ± 0.01^d^	1.15 ± 0.01^d^	1.14 ± 0.07^c^
	9	1.02 ± 0.02^d^	1.01 ± 0.01^e^	1.01 ± 0.01^e^	0.76 ± 0.01^f^
*T*_21_	0	57.24 ± 0.12^a^	57.24 ± 0.12^a^	57.24 ± 0.12^a^	57.24 ± 0.12^a^
	3	57.37 ± 0.44^a^	57.23 ± 0.08^a^	57.25 ± 0.06^a^	57.22 ± 0.10^a^
	6	57.22 ± 0.09^a^	49.76 ± 0.07^b^	49.77 ± 0.10^b^	49.77 ± 0.11^b^
	9	57.26 ± 0.08^a^	49.73 ± 0.11^b^	49.74 ± 0.12^b^	49.76 ± 0.15^b^
*A*_2b_	0	2.60 ± 0.01^k^	2.60 ± 0.01^k^	2.60 ± 0.01^k^	2.60 ± 0.01^k^
	3	3.77 ± 0.05^i^	3.59 ± 0.01^j^	3.98 ± 0.01^h^	3.64 ± 0.02^j^
	6	4.48 ± 0.02^e^	4.33 ± 0.03^f^	4.32 ± 0.01^f^	4.09 ± 0.01^g^
	9	4.66 ± 0.05^d^	5.23 ± 0.01^b^	5.08 ± 0.02^c^	5.34 ± 0.02^a^
*A*_21_	0	97.38 ± 0.03^a^	97.38 ± 0.03^a^	97.38 ± 0.03^a^	97.38 ± 0.03^a^
	3	96.22 ± 0.02^c^	96.43 ± 0.03^b^	96.03 ± 0.04^d^	96.37 ± 0.02^b^
	6	95.54 ± 0.04^g^	95.66 ± 0.04^f^	95.68 ± 0.03^f^	95.90 ± 0.02^e^
	9	95.34 ± 0.03^h^	94.77 ± 0.03^j^	94.93 ± 0.02^i^	94.66 ± 0.01^k^

Note

Values are means ± standard deviations. Values with different superscript letters in the same indicator are significantly different (*p* <.05).

Abbreviation: CLS, cross‐linked starch; CS, corn starch; PS, potato starch; TS, tapioca starch.

**FIGURE 7:**
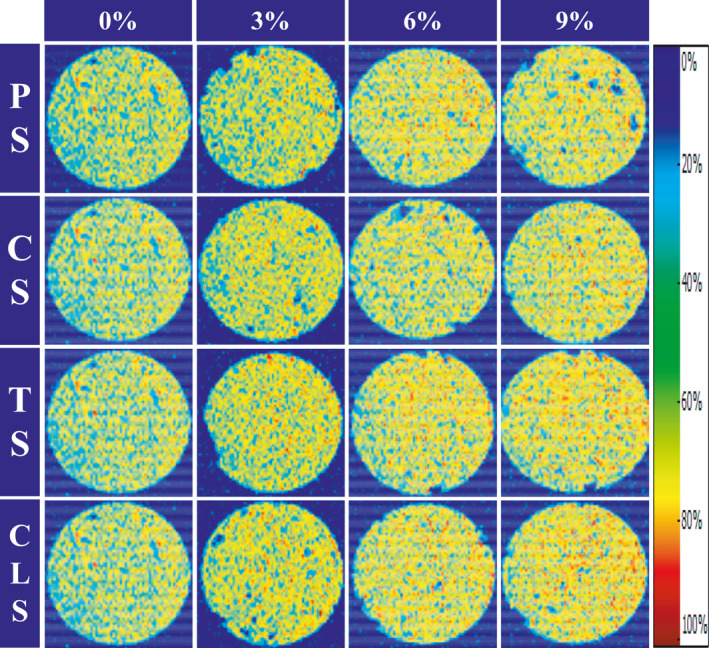
MRI images for shrimp surimi with different types of starch added, PS, potato starch; CS, corn starch; TS, tapioca starch; CLS, cross‐linked starch

MRI is a direct, rapid, noninvasive, nondestructive, and accurate food analysis tool that can reflect the distribution and content of water by measuring the density and distribution of hydrogen protons in the samples. It is considered a powerful tool for visually studying the spatial distribution of water molecules in a food matrix (Cheng et al., 2020). The proton density images show the distribution of water in samples. Generally, if there are more hydrogen protons in a given area, the proton density and pseudocolor images become brighter and redder, respectively (Zhang et al., 2019). As the hydrogen protons move from scattered to dense, the color of the proton spectrum turns from blue to red, that is, the regions with higher water content are red, and those with lower water content are blue (Wang et al., 2016). Figure 7 shows the proton density image of shrimp surimi with different starches added. It can be seen that for the groups with the same type of starch added, the redness of the shrimp surimi image gradually increased with an increase in starch content, thereby increasing the moisture signal. This might be because the addition of starch makes the water distribution of the shrimp surimi sample more uniform, which enhances the ability of the sample to bind water (Luo et al., 2020). In particular, the proton density image of the 9% CLS group was the reddest, indicating that its ability to bind water was the strongest, which may be related to its strong water absorption capacity. This result is consistent with those of WHC and *A*_2b_.

### 3.5 Water holding capacity and water content of shrimp surimi

The WHC affects the supportability of the material, which affects the stability of 3D printing. The effects of the addition of different starches on the WHC of shrimp surimi are shown in Table 4. It can be observed that, for the same type of starch, the WHC of the sample increased with an increase in starch content. When the amount of starch added was 6%, the WHC of shrimp surimi exceeded 89%, which was significantly higher than that of the control group (84.66%) (*p* <.05). This may be because starch has water absorption ability. This result is similar to that of a study by Yang et al., (2014), who found that with the addition of resistant starch, the WHC of grass carp surimi gel increased. WHC reflects the binding status of protein and water molecules in shrimp surimi and the strength of the network structure. Generally, shrimp surimi with good WHC tends to have high elasticity, and the internal water does not leak easily (Rawdkuen et al., 2004), which corresponds to the results of the TPA. For the groups with the same amount of starch added, the WHC of the CLS group was higher than that of other groups, followed by the TS, CS, and PS groups (*p* <.05). This may be related to the extremely strong water absorption capacity of CLS and TS and, to a certain extent, to the poor water absorption capacity of CS and PS. The WHC of shrimp surimi indirectly reflects the compactness of the microscopic network structure of shrimp surimi. The higher the WHC, the stronger the binding capacity of the network structure of shrimp surimi to water and starch, that is, the denser and more uniform the spatial network structure (Liu et al., 2008). Combined with the 3D printing properties, it can be concluded that the printing accuracy and stability of shrimp surimi are correlated with its WHC. The addition of starch increased the WHC of shrimp surimi, which gave the printing products improved supportability and improved the printing accuracy and stability. In brief, suitable WHC is beneficial to improve the 3D printing adaptability of shrimp surimi.

**TABLE 4:** Effects of different types of added starch on the WHC and water content of shrimp surimi

Index	Addition (%)	PS	CS	TS	CLS
WHC (%)	0	84.66 ± 0.40^f^	84.66 ± 0.40^f^	84.66 ± 0.40^f^	84.66 ± 0.40^f^
	3	85.04 ± 0.24^f^	87.86 ± 0.24^e^	88.37 ± 0.88^e^	89.27 ± 0.34^de^
	6	89.07 ± 0.79^de^	89.46 ± 0.12^cde^	89.69 ± 0.95^cde^	91.37 ± 1.17^abc^
	9	90.56 ± 0.32^bcd^	91.89 ± 0.69^ab^	92.53 ± 0.27^a^	93.05 ± 1.01^a^
Water content (%)	0	74.69 ± 0.12^a^	74.69 ± 0.12^a^	74.69 ± 0.12^a^	74.69 ± 0.12^a^
	3	73.33 ± 0.20^b^	73.25 ± 0.24^b^	73.06 ± 0.27^b^	72.90 ± 0.16^b^
	6	72.01 ± 0.35^c^	71.96 ± 0.17^c^	71.83 ± 0.23^c^	71.58 ± 0.28^c^
	9	70.54 ± 0.11^d^	70.43 ± 0.19^d^	70.14 ± 0.16^d^	70.11 ± 0.11^d^

Note

Values are means ± standard deviations. Values with different superscript letters in the same indicator are significantly different (*p* <.05).

Abbreviation: CLS, cross‐linked starch; CS, corn starch; PS, potato starch; TS, tapioca starch.

Water is the most abundant ingredient in shrimp surimi, which directly affects the texture properties of shrimp surimi (Liu et al., 2021), thereby affecting the 3D printing accuracy and stability. The water content of shrimp surimi adding different types of starch was shown in Table 4. From Table 4, there was no significant difference (*p* >.05) in the water content of shrimp surimi adding the same amount of different types of starch, indicating that different types of starch did not affect the water content of shrimp surimi. However, the water content of shrimp surimi gradually decreased (*p* <.05) with increasing the amount of starch. The water mass in shrimp surimi was fixed, when starch was added, the total mass of mixture increased, while the water mass remained unchanged, so the relative water content of mixture decreased. These results explained why the hardness of shrimp surimi adding starch increased and the 3D printing stability increased with increasing the amount of starch.

### 3.6 Principal component analysis

**FIGURE 8:**
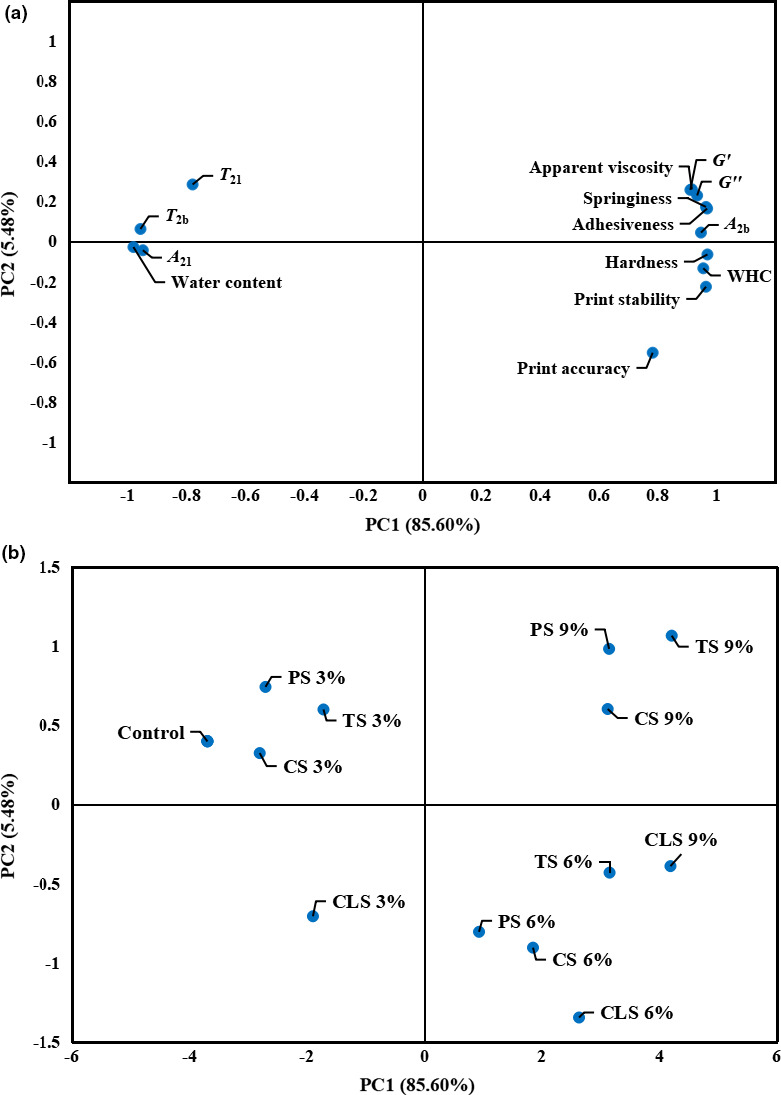
Principal component analysis (PCA) for shrimp surimi with different types of starch added, PS, potato starch; CS, corn starch; TS, tapioca starch; CLS cross‐linked starch. (a) Loadings for the first two principal components; (b) weighed PCA bi‐plot of scores. *G*′, Storage modulus; *G*'', loss modulus; WHC, water holding capacity

Although the starch in shrimp surimi plays a key role in its physical properties, there is currently no objective method to exhibit this effect. In this study, PCA was used to establish the relationship between the amount of different starches, 3D printing properties, and physical properties of shrimp surimi. As a result, a linear combination was obtained by PCA (Sun et al., 2019). It can be observed from Figure 8 that the first two principal components accounted for 91.08% (85.60% PC1 and 5.48% PC2) of the total variance. The eigenvectors of the principal component 1 (PC1) were printing accuracy, printing stability, *G*', *G*'', apparent viscosity, hardness, adhesiveness, springiness, water content, *T*_2b_, *T*_21_, *A*_2b_, *A*_21_, and WHC. It can be seen from Figure 8a that the characteristics of the shrimp surimi were in quadrants 1, 2, 3, and 4. The printing stability and accuracy of the samples were positively correlated with WHC, hardness, adhesion, springiness, *G*', *G*'', *A*_2b_, and apparent viscosity, which were all on the same side of PC1. The printing accuracy and stability were negatively correlated with water content, *T*_2b_, *T*_21_, and *A*_21_, which were in the opposite quadrant of PC1.

Figure 8b shows the score diagram of projections of different treatments using PC1 and PC2 as load factors in a two‐dimensional space. The process inferred to be located in quadrants 1, 2, 3, and 4 was related to variables in the corresponding quadrants. The projection of the samples onto the space of the two first PCs (Figure 8b) showed a difference among shrimp surimi with the addition of different types and amounts of starch. Comparing the score graph (Figure 8b) with the load chart (Figure 8a), it can be observed that the control group without starch added and the groups with 3% starch added were farther away from the printing accuracy and stability, which were located in the different quadrants of PC1. This also proved that the printing accuracy and stability of the shrimp surimi without adding starch or adding a small amount of starch were relatively poor. The printing accuracy, stability, and the groups with 6% or 9% starch added were all in the positive direction of PC1, indicating that these treatments had higher printing stability and accuracy. In addition, all the samples with 6% starch added and the printing accuracy and stability were all in the same quadrant (quadrant 4), indicating that adding the proper amount of starch to shrimp can improve the accuracy and stability of 3D printing products. In addition, the 6% CLS group was the closest to the printing accuracy and stability among all the groups, further verifying that the 6% CLS addition could effectively improve the printing accuracy and stability of shrimp surimi.

## 4 CONCLUSIONS

The addition of starch can effectively improve the 3D printing accuracy and stability of shrimp surimi. The shrimp surimi with 6% CLS had the highest printing accuracy and stability among all the groups studied. Additionally, 6% CLS appropriately increased the *G*', *G*", and apparent viscosity of shrimp surimi, which made it easy to extrude the sample from the nozzle, improved the stacking performance, and solved the problem of broken silk in sample printing. In addition, the hardness, adhesiveness, and springiness of shrimp surimi were increased by the addition of 6% CLS, which increased the supportability and adhesion of the sample and helped the sample maintain its original shape. The results of water characteristic analysis showed that the addition of 6% CLS increased the water binding ability of the sample (especially the binding ability of the bound water), increased the WHC and decreased the water content of the shrimp surimi. In summary, adding 6% CLS can improve the 3D printability of shrimp surimi.

## CONFLICTS OF INTEREST

The authors declare that they have no competing interests.

## References

[fsn32257-bib-0001] Ahmad, M. U. , Tashiro, Y. , Matsukawa, S. , & Ogawa, H. (2007). Gelation mechanism of surimi studied by 1 h NMR relaxation measurements. Journal of Food Science, 72(6), E362–E367. 10.1111/j.1750-3841.2007.00411.x 17995681

[fsn32257-bib-0002] Azam, R. S. M. , Zhang, M. , Bhandari, B. , & Yang, C. (2018). Effect of different gums on features of 3D printed object based on Vitamin‐D enriched orange concentrate. Food Biophysics, 13(8), 250–262. 10.1007/s11483-018-9531-x

[fsn32257-bib-0003] Casas, C. , Martinez, O. , Guillen, M. D. , Pin, C. , & Salmeron, J. (2006). Textural properties of raw Atlantic salmon (*Salmo salar*) at three points along the fillet, determined by different methods. Food Control, 17(7), 511–515. 10.1016/j.foodcont.2005.02.013

[fsn32257-bib-0004] Chen, H. , Xie, F. , Chen, L. , & Zheng, B. (2018). Effect of rheological properties of potato, rice and corn starches on their hot‐extrusion 3d printing behaviors. Journal of Food Engineering, 244, 150–158. 10.1016/j.jfoodeng.2018.09.011

[fsn32257-bib-0005] Cheng, S. , Wang, X. , Yang, H. , Lin, R. , Wang, H. , & Tan, M. (2020). Characterization of moisture migration of beef during refrigeration storage by low‐field NMR and its relationship to beef quality. Journal of the Science of Food and Agriculture, 100, 1940–1948. 10.1002/jsfa.10206 31846075

[fsn32257-bib-0006] Dankar, I. , Haddarah, A. , Omar, F. E. L. , Sepulcre, F. , & Pujolà, M. (2018). 3D printing technology: The new era for food customization and elaboration. Trends in Food Science & Technology, 75, 231–242. 10.1016/j.tifs.2018.03.018

[fsn32257-bib-0007] Dong, X. , Huang, Y. , Pan, Y. , Wang, K. , Sangeeta, P. , & Zhu, B. (2019). Investigation of sweet potato starch as a structural enhancer for three‐dimensional printing of *Scomberomorus niphonius* surimi. Journal of Texture Studies, 50, 316–324. 10.1111/jtxs.12398 30847926

[fsn32257-bib-0008] Eliasson, A. C. (2007). Viscoelastic behaviour during the gelatinization of starch comparison of wheat, maize, potato and waxy‐barley starches. Journal of Texture Studies, 17(3), 253–265. 10.1111/j.1745-4603.1986.tb00551.x

[fsn32257-bib-0009] Fischer, P. , & Windhab, E. J. (2011). Rheology of food materials. Current Opinion in Colloid & Interface Science, 16(1), 36–40. 10.1016/j.cocis.2010.07.003

[fsn32257-bib-0010] Godoi, F. C. , Prakash, S. , & Bhandari, B. R. (2016). 3d printing technologies applied for food design: Status and prospects. Journal of Food Engineering, 179, 44–54. 10.1016/j.jfoodeng.2016.01.025

[fsn32257-bib-0011] Goh, K. S. , Bhat, R. , & Karim, A. A. (2009). Probing the sol‐gel transition of egg white proteins by pulsed‐NMR method. European Food Research and Technology, 228(3), 367–371. 10.1007/s00217-008-0942-7

[fsn32257-bib-0012] Hunt, A. , Getty, K. J. K. , & Park, J. W. (2009). Roles of starch in surimi seafood: A review. Food Reviews International, 25(4), 299–312. 10.1080/87559120903155834

[fsn32257-bib-0013] Jonsson, A. , Sigurgisladottir, S. , Hafsteinsson, H. , & Kristbergsson, K. (2015). Textural properties of raw Atlantic salmon (*Salmo salar*) fillets measured by different methods in comparison to expressible moisture. Aquaculture Nutrition, 7(2), 81–89. 10.1046/j.1365-2095.2001.00152.x

[fsn32257-bib-0014] Kong, W. , Zhang, T. , Feng, D. , Xue, Y. , Wang, Y. , Li, Z. , Yang, W. , & Xue, C. (2016). Effects of modified starches on the gel properties of Alaska Pollock surimi subjected to different temperature treatments. Food Hydrocolloids, 56(1), 20–28. 10.1016/j.foodhyd.2015.11.023

[fsn32257-bib-0015] Kou, T. , & Gao, Q. (2019). A study on the thermal stability of amylose‐amylopectin and amylopectin‐amylopectin in cross‐linked starches through iodine binding capacity. Food Hydrocolloids, 88, 86–91. 10.1016/j.foodhyd.2018.09.028

[fsn32257-bib-0016] Kureshy, N. , & Davis, D. A. (2002). Protein requirement for maintenance and maximum weight gain for the pacific white shrimp. Litopenaeus Vannamei Aquaculture, 204(1–2), 125–143. 10.1016/S0044-8486(01)00649-4

[fsn32257-bib-0017] Liu, R. , Zhao, S. M. , Xiong, S. B. , Xie, B. J. , & Qin, L. H. (2008). Role of secondary structures in the gelation of porcine myosin at different pH values. Meat Science, 80(3), 632–639. 10.1016/j.meatsci.2008.02.014 22063575

[fsn32257-bib-0018] Liu, Y. , Sun, Q. , Pan, Y. , Wei, S. , Xia, Q. , Liu, S. , Ji, H. , Deng, C. , & Hao, J. (2021). Investigation on the correlation between changes in water and texture properties during the processing of surimi from golden pompano (*Trachinotus ovatus*). Journal of Food Science, 86(2), 376–384. 10.1111/1750-3841.15581 33438246

[fsn32257-bib-0019] Liu, Z. , Zhang, M. , Bhandari, B. , & Wang, Y. (2017). 3D printing: Printing precision and application in food sector. Trends in Food Science & Technology, 69, 83–94. 10.1016/j.tifs.2017.08.018

[fsn32257-bib-0020] Liu, Z. , Zhang, M. , Bhandari, B. , & Yang, C. (2018). Impact of rheological properties of mashed potatoes on 3D printing. Journal of Food Engineering, 220, 76–82. 10.1016/j.jfoodeng.2017.04.017

[fsn32257-bib-0021] Luo, H. , Guo, C. , Lin, L. , Si, Y. , Gao, X. , Xu, D. , Jia, R. , & Yang, W. (2020). Combined use of rheology, LF‐NMR, and MRI for characterizing the gel properties of Hairtail surimi with potato starch. Food and Bioprocess Technology, 13, 637–647. 10.1007/s11947-020-02423-y

[fsn32257-bib-0022] Murphy, S. V. , & Atala, A. (2014). 3D bioprinting of tissues and organs. Nature Biotechnology, 32(8), 773–785. 10.1038/nbt.2958 25093879

[fsn32257-bib-0023] Rawdkuen, S. , Benjakul, S. , Visessanguan, W. , & Lanier, T. C. (2004). Chicken plasma protein affects gelation of surimi from bigeye snapper (*Priacanthus tayenus*). Food Hydrocolloids, 18(2), 259–270. 10.1016/S0268-005X(03)00082-1

[fsn32257-bib-0024] Sánchez‐Alonso, I. , Moreno, P. , & Careche, M. (2014). Low field nuclear magnetic resonance (LF‐NMR) relaxometry in hake (*Merluccius merluccius, L*.) muscle after different freezing and storage conditions. Food Chemistry, 153, 250–257. 10.1016/j.foodchem.2013.12.060 24491727

[fsn32257-bib-0025] Sang, Y. , Prakash, O. , & Seib, P. A. (2007). Characterization of phosphorylated cross‐linked resistant starch by 31P nuclear magnetic resonance (31P NMR) spectroscopy. Carbohydrate Polymers, 67(2), 201–212. 10.1016/j.carbpol.2006.05.009

[fsn32257-bib-0026] Severini, C. , Azzollini, D. , Albenzio, M. , & Derossi, A. (2018). On printability, quality and nutritional properties of 3D printed cereal based snacks enriched with edible insects. Food Research International, 106, 666–676. 10.1016/j.foodres.2018.01.034 29579973

[fsn32257-bib-0027] Severini, C. , Derossi, A. , & Azzollini, D. (2016). Variables affecting the printability of foods: Preliminary tests on cereal‐based products. Innovative Food Science & Emerging Technologies, 38, 281–291. 10.1016/j.ifset.2016.10.001

[fsn32257-bib-0028] Steeneken, P. A. M. (1989). Rheological properties of aqueous suspensions of swollen starch granules. Carbohydrate Polymers, 11(1), 23–42. 10.1016/0144-8617(89)90041-6

[fsn32257-bib-0029] Sun, Q. , Sun, F. , Xia, X. , Xu, H. , & Kong, B. (2019). The comparison of ultrasound‐assisted immersion freezing, air freezing and immersion freezing on the muscle quality and physicochemical properties of common carp (*Cyprinus carpio*) during freezing storage. Ultrasonics Sonochemistry, 51, 281–291. 10.1016/j.ultsonch.2018.10.006 30337027

[fsn32257-bib-0030] Wang, L. , Zhang, M. , Bhandari, B. , & Gao, Z. (2016). Effects of malondialdehyde‐induced protein modification on water functionality and physicochemical state of fish myofibrillar protein gel. Food Research International, 86, 131–139. 10.1016/j.foodres.2016.06.007

[fsn32257-bib-0031] Wang, L. , Zhang, M. , Bhandari, B. , & Yang, C. (2018). Investigation on fish surimi gel as promising food material for 3D printing. Journal of Food Engineering, 220, 101–108. 10.1016/j.jfoodeng.2017.02.029

[fsn32257-bib-0032] Whittenberger, R. T. , & Nutting, G. C. (2002). Potato‐starch gels. Industrial and Engineering Chemistry Research, 40(8), 1407–1413. 10.1021/ie50464a015

[fsn32257-bib-0033] Wongsagonsup, R. , Pujchakarn, T. , Jitrakbumrung, S. , Chaiwat, W. , Fuongfuchat, A. , Varavinit, S. , Dangtip, S. , & Suphantharika, M. (2014). Effect of cross‐linking on physicochemical properties of tapioca starch and its application in soup product. Carbohydrate Polymers, 101, 656–665. 10.1016/j.carbpol.2013.09.100 24299823

[fsn32257-bib-0034] Yakubu, P. I. , Ozu, E. M. , Baianu, I. C. , & Orr, P. H. (1993). Hydration of potato starch in aqueous suspensions determined from nuclear magnetic relation studies by oxygen‐17, deuterium, and proton NMR: Relaxation mechanisms and quantitative analysis. Journal of Agricultural and Food Chemistry, 41(2), 162–167. 10.1021/jf00026a003

[fsn32257-bib-0035] Yang, F. , Zhang, M. , Bhandari, B. , & Liu, Y. (2018). Investigation on lemon juice gel as food material for 3D printing and optimization of printing parameters. LWT‐Food Science and Technology, 87, 67–76. 10.1016/j.lwt.2017.08.054

[fsn32257-bib-0036] Yang, F. , Zhang, M. , Prakash, S. , & Liu, Y. (2018). Physical properties of 3D printed baking dough as affected by different compositions. Innovative Food Science & Emerging Technologies, 49, 202–210. 10.1016/j.ifset.2018.01.001

[fsn32257-bib-0037] Yang, H. , & Park, J. W. (1998). Effects of starch properties and thermal‐processing conditions on surimi‐starch gels. LWT‐Food Science and Technology, 31(4), 344–353. 10.1006/fstl.1997.0366

[fsn32257-bib-0038] Yang, Z. , Wang, W. , Wang, H. , & Ye, Q. (2014). Effects of a highly resistant rice starch and pre‐incubation temperatures on the physicochemical properties of surimi gel from grass carp (*Ctenopharyn Odon Idellus*). Food Chemistry, 145(4), 212–219. 10.1016/j.foodchem.2013.08.040 24128470

[fsn32257-bib-0039] Zhang, F. , Fang, L. , Wang, C. , Shi, L. , Chang, T. , Yang, H. , & Cui, M. (2013). Effects of starches on the textural, rheological, and color properties of surimi‐beef gels with microbial tranglutaminase. Meat Science, 93(3), 533–537. 10.1016/j.meatsci.2012.11.013 23273461

[fsn32257-bib-0040] Zhang, H. , Xiong, Y. , Bakry, A. M. , Xiong, S. , Yin, T. , Zhang, B. , Huang, J. , Liu, Z. , & Huang, Q. (2019). Effect of yeast β‐glucan on gel properties, spatial structure and sensory characteristics of silver carp surimi. Food Hydrocolloids, 88, 256–264. 10.1016/j.foodhyd.2018.10.010

[fsn32257-bib-0041] Zhang, L. , Xue, Y. , Xu, J. , Li, Z. , & Xue, C. (2013). Effects of high‐temperature treatment (⩾100 °C) on Alaska Pollock (*theragra chalcogramma*) surimi gel. Journal of Food Engineering, 115(1), 115–120. 10.1016/j.jfoodeng.2012.10.006

[fsn32257-bib-0042] Zhang, Z. , Regenstein, J. M. , Zhou, P. , & Yang, Y. (2016). Effects of high intensity ultrasound modification on physicochemical property and water in myofibrillar protein gel. Ultrasonics Sonochemistry, 34, 960–967. 10.1016/j.ultsonch.2016.08.008 27773327

[fsn32257-bib-0043] Zheng, B. , Wang, H. , Shang, W. , Xie, F. , Li, X. , Chen, L. , & Zhou, Z. (2018). Understanding the digestibility and nutritional functions of rice starch subjected to heat‐moisture treatment. Journal of Functional Foods, 45, 165–172. 10.1016/j.jff.2018.03.041

[fsn32257-bib-0044] Zhou, W. , Yang, J. , Hong, Y. , Liu, G. , Zheng, J. , Gu, Z. , & Zhang, P. (2015). Impact of amylose content on starch physicochemical properties in transgenic sweet potato. Carbohydrate Polymers, 122, 417–427. 10.1016/j.carbpol.2014.11.003 25817686

